# Clinical experience with ipilimumab 10 mg/kg in patients with melanoma treated at Italian centres as part of a European expanded access programme

**DOI:** 10.1186/1756-9966-32-82

**Published:** 2013-10-25

**Authors:** Maresa Altomonte, Anna Maria Di Giacomo, Paola Queirolo, Paolo Antonio Ascierto, Francesco Spagnolo, Emilio Bajetta, Luana Calabrò, Riccardo Danielli, Francesco de Rosa, Michela Maur, Vanna Chiarion-Sileni, Pier Francesco Ferrucci, Diana Giannarelli, Alessandro Testori, Ruggero Ridolfi, Michele Maio

**Affiliations:** 1 University Hospital of Siena, Siena, Italy; 2 San Martino Hospital, National Institute for Cancer Research, Genoa, Italy; 3 Unit of Melanoma, Cancer Immunotherapy and Innovative Therapy, Istituto Nazionale Tumori Fondazione “G. Pascale”, Naples, Italy; 4 Istituto di Oncologia, Policlinico di Monza, Monza, Italy; 5 IRCCS - IRST, Meldola, Forlì-Cesena, Italy; 6 University Hospital, Modena, Italy; 7 Oncology Institute of Veneto, Padua, Italy; 8 Istituto Europeo di Oncologia, Milan, Italy; 9 Statistics, Regina Elena National Cancer Institute, Rome, Italy

**Keywords:** Compassionate use, Expanded access programme, Ipilimumab, Metastatic melanoma

## Abstract

**Background:**

Patients with advanced melanoma are faced with a poor prognosis and, until recently, limited treatment options. Ipilimumab, a novel immunotherapy that blocks cytotoxic T-lymphocyte-associated antigen-4, was the first agent to improve survival of patients with advanced melanoma in a randomised, controlled phase 3 trial. We used data from an expanded access programme (EAP) at Italian centres to evaluate the clinical activity and safety profile of ipilimumab 10 mg/kg in patients with advanced melanoma in a setting more similar to that of daily practice.

**Methods:**

Data were collected from patients enrolled in an ipilimumab EAP across eight participating Italian centres. As per the EAP protocol, patients had life-threatening, unresectable stage III/IV melanoma, had failed or did not tolerate previous treatments and had no other therapeutic option available. Treatment comprised ipilimumab 10 mg/kg every 3 weeks for a total of four doses. If physicians believed patients would continue to derive benefit from ipilimumab treatment, maintenance therapy with ipilimumab 10 mg/kg was provided every 12 weeks. Tumour responses were assessed every 12 weeks using modified World Health Organization criteria and safety continuously monitored.

**Results:**

Seventy-four pretreated patients with advanced melanoma were treated with ipilimumab 10 mg/kg. Of these, 9 (13.0%) had an objective response, comprising 3 patients with a complete response and 6 with a partial response. Median overall survival was 7.0 months (95% confidence interval, 5.3–8.7) and 16.6% of patients were alive after 3 years. Forty-five patients (60.8%) reported treatment-related adverse events of any grade, which were most commonly low-grade pruritus, pain, fever and diarrhoea. Grade 3 or 4 treatment-related AEs were reported in 8 patients (10.8%).

**Conclusions:**

The clinical activity and safety profile of ipilimumab 10 mg/kg in the EAP was similar to that seen in previous clinical trials of ipilimumab in pretreated patient populations.

## Background

Until recently, patients with unresectable stage III and IV (advanced) melanoma faced a dismal prognosis and had limited treatment options [1-4]. However, advances in the understanding of cancer immunology and the molecular pathways involved in melanoma pathogenesis led to exciting developments and new treatments with the potential to provide patients with improved survival. In 2011, two new agents were approved for the treatment of patients with advanced melanoma; ipilimumab, a novel immunotherapy, and vemurafenib, a specific BRAF inhibitor for patients with BRAF^V600^-mutation positive melanoma. These agents have changed the treatment landscape for this difficult to treat disease, with other novel therapeutic approaches on the horizon [5,6].

Ipilimumab is a fully-humanised monoclonal antibody that blocks cytotoxic T-lymphocyte-associated antigen-4, a negative regulator of T cells. This blockade potentiates T-cell activation, proliferation and infiltration into tumours [7]. The antitumour activity of ipilimumab in patients with advanced melanoma has been demonstrated in a number of phase 2 trials [8-12] and in two phase 3 trials; one as monotherapy in patients with pretreated metastatic melanoma at a dose of 3 mg/kg, and the second in combination with chemotherapy in patients with previously untreated metastatic melanoma at a dose of 10 mg/kg [13,14]. Recent updates from clinical trials of pretreated and treatment-naïve patients treated with ipilimumab 10 mg/kg have shown long-term clinical benefit, with some patients surviving at least 5 years [15,16]. Most adverse events (AEs) to ipilimumab reported in clinical trials are low-grade and immune-related and, in most cases, can be managed with appropriate medical therapy, treatment interruption or withdrawal [17-19].

Clinical trials, by virtue of design, include highly-selected patient populations and investigative agents are provided according to tightly-regulated protocols. The ipilimumab Expanded Access Programme (EAP; formerly known as the ipilimumab Compassionate Use Programme) was initiated to provide ipilimumab to patients who were not eligible to receive ipilimumab within clinical trials, therefore providing a real-world perspective on the efficacy, safety and general management of ipilimumab in a setting representative of daily clinical practice. Initially, ipilimumab was provided at a dose of 10 mg/kg; however, the protocol was later amended to allow the use of ipilimumab 3 mg/kg, in line with the approved European label.

Results from 27 patients treated with ipilimumab 10 mg/kg at a single centre in the EAP in Italy have previously been reported [20,21]. Here, we provide long-term follow-up from all patients treated with ipilimumab 10 mg/kg within a participating Italian centre.

## Methods

### Patient population

This was a retrospective analysis of data from patients whose physician requested compassionate use of ipilimumab through the EAP. Patients older than 16 years of age with histologically confirmed unresectable stage III and stage IV skin, ocular or mucosal melanoma were eligible for inclusion in the EAP. Patients must have failed systemic therapy, been intolerant to ≥1 systemic therapy, or had no other therapeutic option available to them. An Eastern Cooperative Oncology Group (ECOG) performance status (PS) of 0, 1, or 2 was required, and an interval of at least 28 days since treatment with chemotherapy, biochemotherapy, surgery, radiation, or immunotherapy was recommended. Patients with asymptomatic brain metastases were allowed. Exclusion criteria included any other systemic therapy for melanoma, concomitant autoimmune diseases or other malignancies, and known HIV, hepatitis B, or hepatitis C infection.

### Study design and data collection

Ipilimumab 10 mg/kg was administered intravenously over 90 minutes every 3 weeks for a total of four doses (induction phase). Eligible patients could receive maintenance therapy with ipilimumab 10 mg/kg every 12 weeks, if tolerated, for as long as the physician believed that benefit would be derived from treatment. Patients who progressed following either stable disease (SD) of ≥3 months’ duration or an initial objective response (partial [PR] or complete response [CR]) were offered retreatment with the same dosing schedule used during the induction phase.

Treatment was discontinued in cases of confirmed disease progression as determined using modified World Health Organization (mWHO) criteria, a related AE necessitating discontinuation of ipilimumab, clinical deterioration (as per protocol), withdrawal of consent, or pregnancy.

Tumour assessments utilising helical (spiral) computerised tomography scans of brain, chest, abdomen, and pelvis, were conducted at baseline and every 12 weeks thereafter. Clinical response was defined according to mWHO criteria as CR, PR, SD or progressive disease (PD). Disease control rate (DCR) was defined as the percentage of patients achieving CR, PR, or SD lasting at least 24 weeks from the first dose of ipilimumab.

Safety was continuously monitored and assessed in all patients who received ipilimumab in the EAP. AEs were graded according to the National Cancer Institute Common Terminology Criteria for AEs, version 3.0.

### Statistical analysis

Data were analysed using descriptive statistics, such as median and range. Progression-free survival (PFS) and overall survival (OS) were estimated using Kaplan–Meier analysis and expressed as median values with corresponding two-sided 95% confidence intervals (CIs).

## Results

### Patients

As part of the EAP, 74 patients with advanced melanoma were treated with ipilimumab 10 mg/kg across eight participating Italian centres. Baseline characteristics of these patients are provided in Table 1. Of the 74 patients treated with ipilimumab, 43 (58.1%) received all four induction doses, 14 (18.9%) received three doses, 5 (6.8%) received two doses and 12 (16.2%) received only one dose. Reasons for discontinuation comprised disease progression (n = 13), death due to disease progression (n = 10), loss to follow-up (n = 5), study drug toxicity (n = 2), and unknown reasons (n = 1). The median number of doses received was four (range: 1–4). Twenty-six patients (35.1%) received maintenance treatment with ipilimumab, with a median number of cycles of two (range: 1–13). Reasons for not receiving maintenance therapy were disease progression (n = 30), death due to disease progression (n = 9), loss to follow up (n = 5) and toxicity (n = 2; 1 patient with grade 2 diarrhoea and nausea, and 1 with grade 1 diarrhoea). As of December 2012, 6 patients were still receiving maintenance therapy. Reasons for discontinuing maintenance therapy were disease progression (n = 14), death due to disease progression (n = 4) and physician decision (n = 1). Two patients with disease progression after four cycles of maintenance therapy were retreated with ipilimumab 10 mg/kg every 3 weeks for a total of four doses; an additional patient with disease progression who did not receive maintenance therapy was retreated with ipilimumab at 3 mg/kg every 3 weeks for a total of four doses.

**Table 1:** Patient characteristics at baseline

**Characteristic**	
Patients, N	74
Gender, n (%)	
Male	46 (62.2)
Female	28 (37.8)
Age, years, median (range)	56 (23–79)
Time from diagnosis, months, median (range)	31 (5–206)
Melanoma diagnosis, n (%)	
Cutaneous	57 (77.0)
Uveal	9 (12.2)
Mucosal	2 (2.7)
Unknown	6 (8.1)
Presence of brain metastases	11 (14.9)
M stage, n (%)	
M0 (unresectable stage III)	2 (2.7)
M1a	16 (21.6)
M1b	2 (2.7)
M1c	53 (71.6)
Unknown	1 (1.4)
ECOG PS	
0	43 (58.1)
1	29 (39.2)
2	2 (2.7)
LDH	
Median (range), units/L	466 (139–4416)
>Upper limit of normal (480 units/L), n (%)	37 (50)
Number of prior therapies for metastatic disease, median (range)	2 (1–5)

*ECOG* Eastern Cooperative Oncology Group, *LDH* lactate dehydrogenase, *PS* performance status.

### Tumour response

Of all treated patients, 69 were evaluable for tumour response (1 patient received three infusions but was not assessed by CT scan, 2 patients were lost to follow-up and 2 patients stopped treatment: 1 due to toxicity and the other for unspecified reasons). Tumour responses according to mWHO criteria are summarised in Table 2. At week 12, 6 patients (8.7%) had an objective response, including 1 patient (1.5%) with a CR and 5 (7.2%) with a PR. Responses to ipilimumab continued to improve beyond week 12: across all treatment phases, a total of 9 patients (13.0%) had an objective response, including 3 patients (4.3%) with a CR and 6 (8.7%) with a PR. Of the 3 patients with an improved response, 1 patient with disease progression improved to CR, and 2 with SD improved to a PR and CR, respectively. The median duration of response has not yet been reached. In total, 22 patients (31.9%) achieved disease control. The median duration of SD was 10 months. Of the 2 patients retreated with ipilimumab 10 mg/kg, 1 regained disease control. The patient retreated with ipilimumab 3 mg/kg also regained disease control, as previously described [21]. Among the 11 patients with brain metastases at baseline, 10 were evaluable for response. Best response to treatment was SD in 2 (20.0%) patients and PD in 8 patients (80.0%).

**Table 2:** Clinical response to ipilimumab 10 mg/kg

**Response**	Responses at the end of induction, week 12 (N = 69)* n (%)	Responses across all treatment phases (N = 69)* n (%)
CR	1 (1.5)	3 (4.3)
PR	5 (7.2)	6 (8.7)
SD	15 (21.7)	13 (18.9)
PD	48 (69.6)	47 (68.1)
ORR	6 (8.7)	9 (13.0)
DCR	N/A	22 (31.9)

* Evaluable patients.

By definition, SD must be of at least 24 weeks duration to be included in the disease control category.

*CR* complete response, *DCR* disease control rate (PR + CR + SD), *N/A* not applicable, *ORR* objective response rate, *PD* progressive disease, *PR* partial response, *SD* stable disease.

### Survival

**Figure 1:**
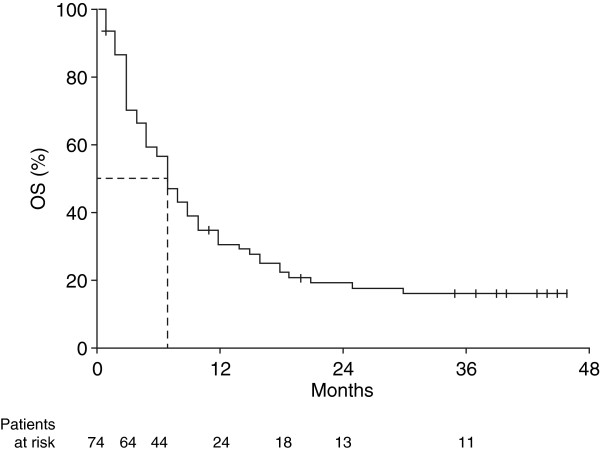
**Kaplan–Meier analysis of OS among 74 patients receiving ipilimumab 10 mg/kg at Italian centres participating in an EAP.***EAP* expanded access programme, *OS* overall survival.

**Figure 2:**
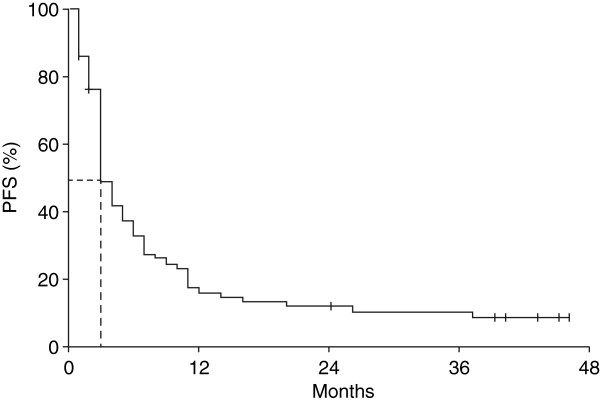
**Kaplan–Meier analysis of progression-free survival among 74 patients receiving ipilimumab 10 mg/kg at Italian centres participating in an EAP.***EAP* expanded access programme, *PFS* progression-free survival.

With a median follow-up of 44 months, median OS was 7.0 months (95% CI, 5.3–8.7) for all patients (Figure 1) and 4.0 months (95% CI, 2.4–5.6) for the 11 patients with brain metastases. Excluding patients with brain metastases did not impact median OS (7.0 months [95% CI, 6.2–9.8]). The 1, 2 and 3-year OS rates were 30.9%, 19.6% and 16.6%, respectively. Ten patients had long-term survival of at least 3 years. The median lactate dehydrohenase (LDH) level for these patients was 280 units/L, and their best response to induction therapy was a PR in 3 patients (30.0%), SD in 6 patients (60.0%) and PD in 1 patient (10.0%). With a median 13 cycles of ipilimumab maintenance therapy, there was an evolution in best response with 2 patients (20.0%) having a CR, and 4 patients (40.0%) each having a PR or SD. Median PFS was 3.0 months (95% CI, 2.3–3.7) for all patients (Figure 2), 3.0 months (95% CI, 2.4–3.6) for the 11 patients with brain metastases and 4.0 months (95% CI, 2.9–5.0) when patients with brain metastases were excluded. As with OS, there was a plateau in PFS after 2 years. Of the 10 patients who survived more than 3 years, only 2 (20.0%) subsequently progressed (after 45 and 57 months, respectively).

### Safety

Of the 74 treated patients, 45 (60.8%) reported at least 1 AE that was considered related to ipilimumab treatment. These are detailed in Table 3. The most commonly reported AEs were pruritus, pain, fever and diarrhoea. Most AEs were grade 1 or 2, with only 8 grade 3 or 4 events reported (by 8 patients [10.8%] in total). Grade 3 or 4 events comprised 2 reports each of diarrhoea and pain; and 1 report each of fever, epigastric pain, elevated aspartate aminotransferase (AST) and pancytopenia. Time to onset of these events was 10–73 days for the diarrhoea, increased AST and pain, and 21 days for fever and pancytopenia. As previously described, grade 4 pancytopenia was successfully managed through the discontinuation of ipilimumab and use of supportive medications (growth factors, transfusions and antibiotics), immunoglobulins and immunosuppressive therapy (cyclosporin) [20].

**Table 3:** Summary of AEs

	**Grade 1**	**Grade 2**	**Grade 3**	**Grade 4**
Patients with a treatment-related AE, n (%)	21 (28.4)	16 (21.6)	7 (9.5)	1 (1.4)
**All events by maximum reported grade, n**				
Any	68	23	7	1
Pruritus	10	3		
Diarrhoea	6	4	2	
Pain	7	2	2	
Fever	8	1	1	
Anaemia	6	1		
Rash	5	2		
Nausea	6			
Asthenia	5			
Cough	2			
Dyspnoea	1	1		
Hyperthyroidism	2			
Hypothyroidism		2		
Infection		2		
Scrotal pain	1	1		
Dermatitis		1		
Oedema		1		
Epigastric pain			1	
Herpes Zoster		1		
Hypocalcemia		1		
Increase in aspartate transaminase/alanine transaminase			1	
Pancytopenia				1
Other*	9			

* Single AEs of grade 1 intensity: penis bleeding, cephalea, confusion, fatigue, constipation, anorexia, inappetence and dysgeusia, flu-like syndrome, ocular Herpes Zoster with oedema and pain.

*AEs* adverse events.

## Discussion

In clinical trials of patients with advanced melanoma, ipilimumab has been shown to provide long-term clinical benefit and have a manageable safety profile [8-14,19]. To evaluate the efficacy and safety profile of ipilimumab in a setting more representative of daily clinical practice, we analysed data from 74 heavily pretreated patients who received ipilimumab 10 mg/kg as part of an EAP in Italy. With an estimated 44 months follow-up across all eight participating centres, median OS was 7.0 months and one-fifth patients had long-term survival of at least 2 years, with approximately 17% of patients alive at 3 years. These findings are consistent with data from clinical trials [22]. In a recent analysis, survival data was pooled from 4846 individual patients treated with ipilimumab within clinical studies or the US EAP. The analysis showed a plateau in OS beginning after approximately 3 years with follow-up of up to 10 years in some patients. Approximately 21% of patients were alive at three years, and survival outcomes did not appear to be impacted by prior therapy, dose or treatment regimen [22]. These data support the durability of long-term survival with ipilimumab and suggest that if patients respond to treatment and are still alive 2 or 3 years after treatment, they have a good chance of achieving long-term tumour control. Indeed, among the 24 patients treated at the University Hospital of Siena in the current analysis, 20% were alive at 4 years, further exemplifying the consistency in long-term survival outcomes [21].

At the first tumour assessment (week 12), 9% of patients in this analysis had achieved an objective response. This is also consistent with previous phase 2 and 3 trials of ipilimumab monotherapy in pretreated populations, with rates ranging from 4–11% with ipilimumab 3 mg/kg and 6–11% with ipilimumab 10 mg/kg [9,12,13].

In previous phase 2 clinical trials of pretreated patients who received ipilimumab 10 mg/kg, median OS has been shown to be approximately 10 months [9,12]. As expected, the median OS reported in this analysis is shorter. Patients had a particularly poor prognosis and in most cases had failed to respond to one or more prior treatments for metastatic disease. Their disease was therefore very advanced. Indeed, most patients (72%) had M1c disease at the time of enrolment. Previous studies have highlighted poor PS, presence of visceral disease, brain metastases, elevated LDH and disease M-stage as statistically significant prognostic factors of poor OS [2,23]. Interestingly, in this analysis, LDH at baseline was lower among patients who survived more than 3 years than for all treated patients: median 280 units/L (range: 59–913) vs 466 units/L (range: 139–4416). Considering the median OS, tumour responses, and 3-year survival rate of patients analysed here, the efficacy of ipilimumab in a real-world setting appears consistent with that observed in selected clinical trial populations.

Beyond week 12, the percentage of patients experiencing an objective response increased. This apparent evolution in responses may reflect the indirect, immune-mediated mechanism of action of ipilimumab. Because it can take time to build an immune response against a tumour, clinically measurable antitumour effects may occur over weeks to months and can be observed after the appearance of new lesions or an initial increase in tumour volume, or in the form of a delayed, slow, steady decline in total tumour volume [14,24,25]. In most cases, clinical benefit with ipilimumab comprised durable SD, which is often the predominant response of patients receiving ipilimumab. In clinical trials, survival outcomes among patients with SD are similar to those of patients with an objective response to ipilimumab [26], suggesting that SD is a meaningful clinical outcome for patients treated with this agent. This observation that most patients seem to obtain a state of tumour control rather than one of complete tumour eradication gives credence to the concept that ‘clinical cure’ or long-term cancer containment is possible, and requires induction of an antitumour immune response [27].

The AE profile seen in patients receiving ipilimumab in the EAP was consistent with that reported in previous clinical trials of ipilimumab 10 mg/kg, with most events being dermatological or gastrointestinal in nature [9,11,12]. Among 155 patients treated with ipilimumab 10 mg/kg in a phase 2 trial, 84% of patients had treatment-related AEs of any grade, with one treatment-related death resulting from liver failure [9]. In our analysis, 61% of patients had treatment-related AEs of any grade and there were no treatment-related deaths. Rates of grade ≥3 AEs appeared similar to those seen in previous clinical trials of ipilimumab 3 mg/kg, but were lower than has been observed in clinical trials of ipilimumab 10 mg/kg [8,9,11-13]. In a trial of ipilimumab 0.3, 3 and 10 mg/kg in pretreated patients with advanced melanoma, the rate and severity of AEs increased with increasing dose; therefore the lack of high-grade events in this analysis is perhaps surprising, although the retrospective nature of this analysis may have influenced the number of AEs reported. Prompt recognition of symptoms and appropriate management are essential to minimise life-threatening complications from ipilimumab. A decrease in the percentage of patients requiring intervention for bowel perforation from 0.9% to 0.5%, for example, was thought to be due to the introduction of management guidelines established over the course of ipilimumab’s clinical development [28]. It is possible, therefore, that an increased awareness of the specific AEs associated with ipilimumab, together with the implementation and consistent use of established treatment algorithms, may have contributed to the safety profile observed in this analysis. The availability of these treatment algorithms also means that, after thorough education on the appropriate management methods, ipilimumab 10 mg/kg can be administered by community-based clinicians, thus extending its use beyond specialised treatment centres.

## Conclusions

Results from this analysis of heavily pretreated patients with advanced melanoma who received ipilimumab 10 mg/kg as part of an EAP in Italy suggest that the clinical activity and safety profile of ipilimumab in a real-world setting is similar to that observed in clinical trials. Importantly, long-term survival benefits were observed in patients who had a particularly poor prognosis and had failed to benefit from prior therapy, with some patients surviving at least 3 years from the start of ipilimumab treatment. Although further studies are needed to establish which dose of ipilimumab (3 or 10 mg/kg) will provide patients with the most appropriate risk to benefit ratio, this analysis shows that in the daily-clinical setting, ipilimumab 10 mg/kg has clinical activity in patients with advanced melanoma with manageable side effects.

## Competing interests

Paolo Ascierto has served in a consultancy/ advisory role for Bristol-Myers Squibb (BMS), Merck Sharp & Dohme (MSD), Roche-Genentech, GlaxoSmithKline (GSK), Amgen and Celgene; has received research funding from BMS, and honoraria from BMS, MSD, Roche-Genentech and GSK. Paola Queirolo has served in a consultant or advisory role for BMS, GSK and Roche-Genentech. Riccardo Danielli has received honoraria from BMS for meeting presentations. Vanna Chiarion-Sileni has had an advisory role for BMS, GSK, MSD and Roche-Genentech. Pier Francesco Ferrucci received honoraria from BMS for participation in a speaker training programme. Michele Maio has had an advisory role and received funding for communication programs from BMS, Roche-Genentech and MSD and has received research funding from BMS. None of the other authors have any conflicts of interest to disclose.

## Authors’ contributions

All authors made substantial contributions to the acquisition and interpretation of data. Statistical support was provided by Dr Giannarelli. All authors were involved in drafting the article or revising it critically for important intellectual content and provided final approval of the version to be published.
